# New Business Structures Creating Organizational Opportunities and Challenges for Work Disability Prevention

**DOI:** 10.1007/s10926-016-9671-0

**Published:** 2016-10-04

**Authors:** Kerstin Ekberg, Glenn S. Pransky, Elyssa Besen, Jean-Baptise Fassier, Michael Feuerstein, Fehmidah Munir, Peter Blanck, Hopkinton Conference Working Group on Workplace Disability Prevention, Benjamin C. Amick, Johannes R. Anema, Elyssa Besen, Peter Blanck, Cécile R. L. Boot, Ute Bültmann, Chetwyn C. H. Chan, George L. Delclos, Kerstin Ekberg, Mark G. Ehrhart, Jean-Baptiste Fassier, Michael Feuerstein, David Gimeno, Vicki L. Kristman, Steven J. Linton, Chris J. Main, Fehmidah Munir, Michael K. Nicholas, Glenn Pransky, William S. Shaw, Michael J. Sullivan, Lois E. Tetrick, Torill H. Tveito, Eira Viikari-Juntura, Kelly Williams-Whitt, Amanda E. Young

**Affiliations:** 1 Division of Community Medicine, Department of Medical and Health Sciences, Linköping University, 581 83 Linköping, Sweden; 2 Liberty Mutual Research Institute for Safety, Hopkinton, MA USA; 3 University of Massachusetts Medical School, Worcester, MA USA; 4 Claude Bernard University Lyon 1, Lyon, France; 5 Uniformed Services University of the Health Sciences, Bethesda, MD USA; 6 School of Sport, Exercise and Health Sciences, Loughborough University, Loughborough, UK; 7 Burton Blatt Institute, Syracuse University, Syracuse, NY USA

**Keywords:** Alternate work arrangements, Disability, Employers, Research priorities

## Abstract

*Purpose* Flexible work arrangements are growing in order to develop resource-efficient production and because of advanced technologies, new societal values, changing demographics, and globalization. The article aims to illustrate the emerging challenges and opportunities for work disability prevention efforts among workers in alternate work arrangements. *Methods* The authors participated in a year-long collaboration that ultimately led to an invited 3-day conference, “Improving Research of Employer Practices to Prevent Disability,” held October 14–16, 2015, in Hopkinton, Massachusetts, USA. The collaboration included a topical review of the literature, group conference calls to identify key areas and challenges, drafting of initial documents, review of industry publications, and a conference presentation that included feedback from peer researchers and a roundtable discussion with experts having direct employer experience. *Results* Both worker and employer perspectives were considered, and four common alternate work arrangements were identified: (a) temporary and contingent employment; (b) small workplaces; (c) virtual work/telework; and (d) lone workers. There was sparse available research of return-to-work (RTW) and workplace disability management strategies with regard to alternate work patterns. Limited research findings and a review of the grey literature suggested that regulations and guidelines concerning disabled workers are often ambiguous, leading to unsatisfactory protection. At the workplace level, there was a lack of research evidence on how flexible work arrangements could be handled or leveraged to support RTW and prevent disability. Potential negative consequences of this lack of organizational guidance and information are higher costs for employers and insurers and feelings of job insecurity, lack of social support and integration, or work intensification for disabled workers. *Conclusions* Future studies of RTW and workplace disability prevention strategies should be designed to reflect the multiple work patterns that currently exist across many working populations, and in particular, flexible work arrangements should be explored in more detail as a possible mechanism for preventing disability. Labor laws and policies need to be developed to fit flexible work arrangements.

## Introduction

The twenty-first century labor market and the organization of work are undergoing continuous change, driven by efforts to increase resource-efficient production, the evolution of technology and resources, and an aging and increasingly diverse workforce. Often workplace changes are associated with down-sizing or right-sizing to reduce staffing levels, work intensification, an increase in the use of temporary employment contracts to handle precarious work, multi-skilling and flexibility in tasks among employees, and outsourcing of work tasks, such as to call centers based in countries with relatively low salary rates [1, 2]. Globalization has further intensified economic integration, increased the intensity of competition among companies and provided greater opportunities to restructure, downsize, and outsource work to subcontractors or lower-wage countries. Globalization has also opened up opportunities for a more diverse labor supply [1]. A recent phenomena is the so-called “gig economy,” or on-demand employment where workers are considered as independent contractors with limited or no societal protection. The development is, according to Virtanen et al. [3], assumed to follow a core-periphery structure. The core employees with relatively secure labor market status are surrounded by sectors of a “buffer work force” with various types of unstable and insecure work arrangements. Thus, work is being redefined by advanced technologies, and changing societal values and demographics [4].

The expansion of temporary and contingent employment, in particular, reflects individual and employer demands for increased flexibility in working patterns [5, 6]. These structural changes in the labor market have implications for how workplaces are organized, the working conditions, variation in work demands placed on employees, and opportunities to implement workplace accommodations for workers with disabilities or for those who may become disabled in the future [5]. The consequences of the changes in the labor market and implications for special categories of workplaces with regard to work disability prevention (WDP) research and return-to-work (RTW) strategies is currently unknown. Work disability is in this context defined as an impairment interfering with work. Most workplace research conducted on disability prevention and RTW has focused on “conventional” workplaces, often large organizations, partly to recruit large samples, but also because these types of workplaces have been the “gold standard” for much of the last century. There is comparably less knowledge on less typical workplaces and work conditions, and on their opportunities and incentives for work disability prevention.

The evolving labor market provides opportunities for workers with competitive work skills (e.g. high education, IT competence, social skills) and the ability to be flexible, but it also creates challenges for RTW strategies, especially for vulnerable groups, such as lower-educated, immigrants, chronically ill, and individuals with disabilities. The consequences for workers include growing job insecurity and work intensification [2]. Women, older and younger workers, and migrant workers tend to be concentrated in particular market sectors and jobs with precarious employment arrangements and non-standard working-times. Workers in restructured workplaces are more likely to report higher exposure to psychosocial workplace risks, higher levels of behavioral health disorders, such as depression, anxiety, and sleep problems, higher levels of work absenteeism and higher “presenteeism,” and related physical and psychosocial risks.

Typically, employment and labor laws and policies are designed mostly for the traditional, full-time labor force which can create RTW difficulties for special categories of workplaces and workers, leaving gaps in regulations and resources within or outside of the workplace that would support disability prevention and RTW. The UN Convention on the Rights of Persons with Disabilities, article 27, states “the right of persons with disabilities to work, on an equal basis with others…including those who acquire a disability during the course of employment”. Appropriate steps to implement these rights include providing vocational guidance programs, placement services and vocational and continuing training, reasonable accommodation in the workplace and return-to-work programs for persons with disabilities. The Convention is intended as a human rights instrument with an explicit, social development dimension and reaffirms that all persons with all types of disabilities must enjoy all human rights and fundamental freedoms [7].

With a goal of improving future research of employer disability prevention strategies, the authors participated in an invited 3-day conference, “Improving Research of Employer Practices to Prevent Disability,” held October 14–16, 2015, in Hopkinton, Massachusetts, USA. Methods and general proceedings of the conference are described in the introductory article to this special issue [8]. The authors of the present article represented a sub-group within the conference tasked with understanding the state of the science with respect to the changing nature of work and its implications for future research and practice in employer-based work disability prevention efforts. We were asked to review the applicable scientific literature, assess its impact for employer decision-making, compare recommendations with that of the employer-directed grey literature, contrast key conceptual and theoretical frameworks, and recommend future research priorities. In this article, we present the results of our research and conference discussions about these challenges from the employer´s and the worker´s perspectives and suggest research priorities for interventions involving structural and organizational changes, as well as workplace conditions and employment security in relation to work disability prevention. We selected four of the most common alternate work arrangements as the focus of our analysis: (a) temporary and contingent employment; (b) small workplaces; (c) virtual work/telework; and (d) lone workers.

## Temporary Work Arrangements (TWA)

One of the key features of labor market developments over the last 25 years has been the increase in the share of temporary and contingent employment (temporary work arrangements [TWA]) in most industrially advanced countries and also in emerging countries [9]. In the US, TWA has more than doubled between 1990 and 2008, with an increase from 1.1 to 2.3 million workers. TWA is increasingly used as a strategic alternative to meet temporary, but also long-term staffing needs [10]. Individuals may value temporary jobs as a means of entering the labor market and securing an immediate source of income, while gaining work experience and skills to move up the job ladder, or to maximize their work flexibility [11, 12]. These positive aspects are cited primarily by higher qualified employees who voluntarily choose TWA employment [12]. Many temporary workers in Europe move into a permanent job within 2 years, while up to one-fourth of temporary workers become unemployed [13].

A critical question in research on associations between temporary employment and health is how to measure temporary employment (e.g. on-demand employment, time-limited, limited to a task). Another issue concerns reasons for temporary employment, e.g. voluntary, ill health, or due to other conditions in the life situation. Some studies indicate, however, that temporary agency workers are often at greater risk of injury than permanent workers [14]. There are several potential reasons for this trend; for example, temporary agencies may not adequately supervise or understand the work conditions of their client employer, workers may be unfamiliar with equipment, processes, and other conditions at the workplace, they may be less likely to receive workplace accommodations if disabled, and they may be less likely to report workplace hazards because of their economic insecurity. TWA workers have more tendency to injury or disability as they often receive no orientation/training and no safety training as the permanent workers receive. TWA workers are also often treated differently than permanent workers by supervisors and co-workers. Due to the short-term nature of the temporary employment relationship, it is often not possible to ensure a best-fit between workers and the jobs they are expected to perform. Temporary workers are overrepresented in smaller firms, in hazardous occupations with inferior working conditions, and often with poor or little compliance with employment regulations. Temporary work agencies also have limited options or no system for providing modified work or accommodations after injuries, and complex employment relationships create uncertainty about liability for injury and RTW [15, 16].

The possibilities for temporary agency workers to RTW at their last employer is limited, as they have no permanent workplace to return to when disabled or sick-listed. Vocational rehabilitation and RTW guidance for this group is not well organized [17–19] as there is often little or no economic support for such employees. These workers also experience a high risk of being replaced when reporting injuries. One study reported that almost half of injured temporary agency workers were offered no further placements after lodging workers´ compensation claims, compared to 14 % of direct hired temporary workers [16]. Reviews by Ferrie et al. [20] and Virtanen et al. [3] showed that temporary employment was associated with poor mental health. Waenerlund et al. [21] used trajectory analysis to measure associations between labor market attachment over 12 years, and health. The probability of psychological distress was higher in groups with different degrees of non-permanent labor market attachment during the time period, as temporary employments or unemployment, the group with least attachment had the worst health status. The poorly attached workers were also more likely to have other burdensome life- and economic factors contributing to psychological distress. Temporary employment has also been associated with increased mortality [22]. Financial hardship and job insecurity are also related to rates of illness.

Conditions for RTW and temporary work were explored by Ervasti et al. [23] in a Finnish cohort study. In work disability due to depressive disorders, temporary employment was associated with slower return to work, especially among older workers and those with lower levels of education. A participatory RTW program involving the disabled and sick-listed worker, a labor expert from the Social Insurance Agency, an independent RTW coordinator, and the use of a vocational rehabilitation agency to find a suitable workplace, were shown to be effective interventions to facilitate work resumption for temporary agency workers and unemployed in a Dutch study [18]. The intervention was more costly than usual care, but as it enhanced work resumption it generated a net socioeconomic benefit [19]. The program has similarities with the evidence-based model Individual Placement and Support (IPS) [24] for persons disabled with serious mental illness, where the workplace is central for successful RTW. In a process evaluation of a participatory program, van Beurden et al. [25] found that timely placement in a suitable temporary workplace, a key feature of the program, was difficult to achieve because of limited availability of appropriate placements. Audhoe et al. [26] developed and evaluated an adapted return to work guideline to be used by physicians for disabled and sick-listed unemployed and temporary employed workers with minor psychological problems. Since no employer was available for the target group, vocational rehabilitation agencies and labor experts were engaged in providing guidance, which was found to be useful for the physicians. However, success of these types of guidelines is dependent on integrating the unique aspects of a particular jurisdiction and resources of the country where it is used, since countries differ in procedures and resources for return to work. Some European countries, such as Belgium, Germany, and the Netherlands, have special policies for temporary work agencies, which are subsidized for placing long-term unemployed or other hard-to-employ workers (e.g. older workers in the Netherlands) into temporary jobs [13]. Preliminary evaluation of the success of subsidized temporary employment in helping to employ disadvantaged groups is reported to be encouraging [27].

## Small and Medium-Sized Enterprises (SMEs)

Small and medium-sized enterprises (SMEs) account for over 95 % of firms, 60–70 % of employment, and generate a large share of new jobs around the globe. The majority of SME’s are small businesses (<50 employees). Most SME jobs are in the service sector, which account for two-thirds of economic activity and employment in OECD countries [28]. Smaller firms are found particularly in wholesale and retail trade, the hotel and restaurant business, communications and business services, and construction. Small companies often have lower levels of disability and sickness absence compared to large companies, but work-related accidents occur relatively more often in small enterprises [29].

SME employers often experience conflict between economic and time pressures and the responsibilities in the sick-leave and RTW process. Haslam et al. [30] examined factors influencing investment in health and safety, and the perceptions of costs due to injuries and illness among SMEs and larger organizations. Most of those reporting from SME’s were uncertain about their costs for work-related illness. Only 10 % of SMEs reported that occupational injuries represented a substantial cost to their business, compared to 56 % of large organizations.

Firm size also is inversely related to duration of disability, and RTW rates are lower in small workplaces. SME’s are less likely to have RTW programs and policies [31], and injured workers are more likely to find re-employment in other workplaces or remain unemployed [15]. Many employers consider it not financially viable to retain employees who cannot return to their initial work assignments [32]. Contact between the employer and the disabled worker on sick-leave often is ad-hoc and determined by the pre-injury quality and social strength of their relationship.

Workplace accommodations may be, but need not necessarily be, more costly to implement in SMEs, due to the lack of overall resources [6]. Andersen et al. [29] found that offering modified work in SME’s typically depended on whether the enterprise already had varied work tasks available. Offers of modified work were dependent on how much the owner valued the injured worker’s experience for the firm, such that job attachment and a good relationship between the owner and the employee made modified work more likely. Similar findings of selective offers of work modification were reported by Seing et al. [33]. In general, SME owners have relatively less knowledge of possibilities for financial and practical support for early return to work initiatives. In an interview study [34], managers of SME’s expressed the need for additional support to facilitate RTW. They had limited or no experience of the process and did not have documented RTW policies. They were willing to make occupational adjustments to support return to one’s usual job, but found it difficult to provide accommodated work tasks.

Small enterprises (<15–20 workers) typically are exempt from legal and policy RTW provisions, for example, in some Canadian provinces, they are not required to have safety committees or to re-employ injured workers [35]. Similar exemptions are found in the U.S. and other countries [5]. Vertical and horizontal subcontracting arrangements are common among SME’s; for example, in construction work, and lead to unclear employer responsibilities. Some SME’s that are high technology firms working internationally outsource hazardous work. Eakin et al. [36] concluded that the diversity and changing nature of SMEs constitutes an important upstream dimension of the OHS problem in small workplaces. In countries where occupational health services are not legally prescribed, fewer SME’s use OHS compared to larger firms.

In a systematic review of the quantitative and qualitative literature, MacEachen et al. [35] conclude that research on OHS interventions in small businesses is heterogeneous in terms of types of interventions implemented, quality of study designs and outcomes measured. They found moderate level of evidence for the effect of OHS interventions on environmental exposure, behavior, attitudes and beliefs and health, but no intervention had negative effects. They conclude that interventions at SME’s need tailoring with regard to the legislative context as some small businesses are exempt from some aspects of OHS legislation, consideration of the social norms about risks and how to respond to them, and tailoring of interventions to sectors [35]. Industry, construction or biotechnology sectors for example have different environmental risks, different types of employment contracts and different levels of education regarding risk handling among the work force, and may therefore also have different opportunities for WDP and offers of workplace accommodations to facilitate RTW.

Another type of intervention was presented as a consequence of one German study [31], where representatives of 1 441 SME’s with 1–250 employees were interviewed. Only one third knew about WDP and the legal obligations for WDP. Only half had a system to collect data about health-related absenteeism. They found that SME’s have a need for consultation in cases of illness and WDP. This has led to implementation of a national project “Gesunde Arbeit” in which consulting structures are established for SME’s. Similar network structures are lacking in many countries, but they are probably one way to improve WDP in SMEs. In the UK, the most significant support for small business employers comes in the form of ‘Access to Work’ (AtW): a labor-market intervention that provides grants to employers which can be used to pay for practical support for staff that have a disability, health or mental health condition. The types of support covered by AtW grants include the purchase of special equipment, a support worker to help disabled staff members in the workplace, and fares to work for staff who cannot use public transport [37].

## Virtual Workplaces: Telework

Virtual or distanced work is increasing, based on employers’ interests in reducing fixed costs and increasing employee performance and retention [5]. Virtual work often is referred to as telework, defined as …“a flexible work arrangement whereby workers work in locations, remote from their central offices or production facilities, with no personal contact with co-workers, but the ability to communicate with co-workers using Information and Communication Technology (ICT)” [38]. Regular work-at-home, among the non-self-employed population, has grown in the US by 103 % since 2005 to 6.5 % in 2014. This represents the largest year over year increase since before the recession, 2.5 % of the workforce now work from home at least half the time [39]. In Europe in 2005 the overall average proportion of employees involved in telecommuting/telework was about 7 % for the entire EU27, with considerable differences between countries and between occupations. Telework is most prevalent in management, sales, professional and office jobs. Teleworkers are usually highly educated, often in supervisory position and are working long weeks according to Statistics Netherlands [40]. Employers can save considerable amounts of money if using telework, savings come from increased productivity, reduced real estate costs and lower absenteeism and turnover, according to Benefits Canada [41].

Working out of the office environment can be a challenge for some people. It may also change the contact between colleagues and between employees and firms, leading to a loss of corporate affiliation. Other inevitable risks are increased work stress, poor attention to ergonomics, and the loss of work and life boundaries. There is only limited research on teleworkers’ health and well-being [42] and evidence-based knowledge of the effect of teleworking on sickness absence and well-being is still lacking [43]. Steward found in an interview study of teleworkers [44] that the work may be characterized by spatial and temporal flexibility, which helped workers to integrate work and family roles. However, teleworkers found no spatial or symbolic boundaries in which to be “ill.” Few physical problems constituted a legitimate reason for not working, the opportunity to work flexible hours enabled teleworkers to mask periods of illness and perhaps longer-term disabilities from employers. The study showed that teleworkers were working longer into illness and sooner in convalescence. With regard to work disability prevention, teleworkers had difficulties filing claims for sickness absence and identifying opportunities for sick leave. They experienced increased personal responsibility for their occupational health and safety and their employers took little interest in their working conditions; for example, only half received a health and safety inspection of their home office. Another, and more positive example was provided by St. George and colleagues [45], who compared tele-nursing from home and tele-nursing in a health call center and found a number of advantages for tele-nursing from home, including fewer sick leave days. In contrast to many other virtual workplaces, the tele-nurses were provided with adequate education, full technological and software facilities and ready access to supervision and continuing education.

Virtual workplaces provide increased opportunity for persons with disabilities to obtain a job. For workers with disabilities home-based telework provides possibilities to access employment unhampered by physical limitations, workplace accessibility, transportation needs and interpersonal problems at a workplace [46]. One of the common reasons for employers to adopt telework programs for disabled workers is to retain highly skilled workers. In one synthesis report [47], successful support strategies for disabled workers were skills-matching, one-to-one coaching and support including assistance in mastering computer operations, development of a home office, and general guidance for job retention. Alternative work options benefitting workers with disabilities included initial part-time work that could lead to full-time work, provision of supplemental income, and transitional work opportunities by learning the norms and culture of service work. The synthesis report suggested establishing intermediary telework organizations with expertise in effective telework employment models and providing training and support for workers with disabilities, and the potential for a positive impact on RTW for disabled workers.

Telework often takes place in the home, but also may occur in remote contexts [48]. The disadvantages of telework may include social isolation. Bentley et al. [48] found that organizational support, such as the degree to which employees believe that their organization values their contributions and cares about their well-being, was related to job satisfaction, to reduced psychological strain and to perceptions of reduced social isolation. They conclude that opportunities for regular face-to-face social interaction with supervisors and co-workers may reduce isolation among teleworkers.

Some researchers question whether telework as an accommodation for workers with disabilities runs counter to the objectives of mainstream inclusion and accessibility as set out in the Americans with Disabilities Act (ADA) and similar legislation [49, 50]. It is argued that telework arrangements may result in increased social exclusion and diminished social capital for workers with disabilities if not actively addressed [51]. Telework accommodations for workers with disabilities often remove physical barriers for participation in the labor market, while the negative side may include isolation and limited opportunities for advancement [49].

## Lone Workers

Lone workers are those who work by themselves without close or direct supervision, such as self-employed, people working alone in premises, people who work from home (teleworkers), people working outside normal hours and who are usually not supervised closely [52]. Other examples of lone workers are workers in small shops, home-workers, construction workers, agricultural workers, and service workers.

In the UK, almost half (46 %) of people in full time employment count themselves as lone workers [53]. Lone work is more common among immigrant workers than natives. Among different labor market branches it is common in construction and transport. Truck drivers represent the second most prevalent lone worker occupation for men in Canada and in other countries. The prevalence of lone working is likely to increase in most countries due to the organizational and technological changes involving virtual workplaces and temporary employments, and new labor laws promoting self-employment.

Most intervention studies related to this group of workers have focused on life style factors such as weight reduction [54]. However, different groups of lone workers are exposed to different health threats. Possible risks are violence and aggressions, occupational risks such as slips, falls, and personal wellbeing and health risks, and lack of social integration. McDonough et al. [55] found in a focus group study of truck drivers´ view on health that the dominant themes were stress and time pressure due to competitiveness of the business, perceived lack of power in the relationship with customers, unhealthy lifestyle behaviors due to workplace demands, missing the family and long working hours. There are guidelines for employers and safety representatives regarding lone working [56, 57], but less research on WDP interventions. Guidelines suggest minimizing lone work by organizational means, using communication technology, regular review procedures of work situation, re-organization, and training of workers and supervisors.

## Special Workplaces and Work Disability Prevention: Research Challenges

The conclusions of this paper are not based on a systematic review of scientific and grey literature, as there is very limited amount of scientific literature with regard to WPD and RTW for the special work conditions discussed herein. Temporary employment contracts, small and medium sized workplaces, telework and lone work are all work situations that have existed for many years, yet they are considered as new in some respects. It is not unreasonable, however, to label these workplaces “new,” as working life is changing faster now than ever before, by the use of modern technology. In parallel, the qualification demands on many workers are changing to include higher levels of cognitive and social skills, flexibility and ability for continuous learning. These demands are stimulating and rewarding for a large number of people, while those who have less personal resources, for example short or no education, ill health, another native language, a strenuous economic situation, may have large challenges in meeting the labor market demands of today. These groups may often end up in precarious work situations with looser employment relations linked to greater levels of job insecurity, and thus more challenges in RTW.

The potential challenges for disability management in nonconventional work arrangements are outlined in Table 1, based on seven evidence-based guidelines by Institute of Work and Health (2007), and supplemented with an eighth challenge, Labor laws and policies.

**Table 1:** Potential challenges for disability management in nonconventional work arrangements

RTW principles	Challenges and opportunities to provide RTW assistance			
	Temporary work arrangement	Small and medium enterprise	Telework/work from home	Lone worker
1. Strong organizational commitment to health and safety	Employer feels less long-term obligation and liability for temporary worker	Workplace support occurs more organically, but organization may be unfamiliar with RTW strategies	Limited access to organizational support	Limited access to organizational support
2. Routine offer of modified duty to facilitate early RTW	Extensive job modification efforts may appear to have little return on investment for a worker with limited tenure and job skills	Job demands may be more flexible, but survival of company depends on maximal individual productivity	Injured or ill worker may have access to greater leeway and flexibility, but medical restrictions may be difficult to enforce and alternate tasks difficult to arrange	No opportunities for co-workers to provide occasional assistance, and fewer opportunities for job leeway and flexibility
3. Support coordination of RTW while not disadvantaging others	Worker may not have established trust and rapport with co-workers	Job modifications and special RTW arrangements may seem intolerable in a small working group	Co-workers may not understand the nature of work limitations if the ill or injured worker is working off-site	Limited access to others to provide RTW coordination and follow-up
4. Supervisors trained and included in RTW planning	Injured or ill worker may not have a designated permanent supervisor on-site to rely on	Relationships with supervisors may be more firmly established, but supervisor unlikely to have RTW training	Relationships with supervisors may be less firmly established, and worker frustrations may not be realized by supervisor	Supervisory role is diminished or distant
5. Early and considerate contact with injured/ill worker	Injured or ill worker may have fewer social ties and close colleagues for advice and support	SMEs may have stronger personal ties with workers to facilitate communication and support	Need for communication may be unclear if worker is off-site. No opportunity for face-to-face empathy and support	Regular communication with the organization may not be routine
6. Designated RTW coordinator	Organizational responsibility for communication and follow-up may be diluted or managed by a third party	SMEs may be less likely to have a designated RTW coordinator with relevant training and methods	RTW coordinator may be less effective by telephone and unaware of ergonomic challenges at home	RTW coordinator may be unaware of job demands
7. Communication between employer and healthcare provider	Injured or ill worker may rely on healthcare providers exclusively for RTW planning and guidance	SMEs have fewer ties with designated health care providers, but more direct communication with supervisors might be feasible	Healthcare provider may be unlikely to have ties with employer or knowledge of work demands	Healthcare provider may be unlikely to have ties with employer or knowledge of work demands
8. Labor laws and policies	Uncertain liability for injury and RTW	Exempt from legal and policy RTW provision	Difficulties in claiming sickness absence	Normal regulations and employer responsibility

*RTW* return to work

At the societal or structural level, involving the legislative context and jurisdictions and regulations regarding benefit claims, studies report difficulties for those employed with temporary work contracts, and for teleworkers and lone workers. Research is needed to disentangle the employer responsibilities for WDP and RTW for these work arrangements and for disabled workers. The possibilities for an intermediary organization providing support in work environment and benefit claim issues need to be further explored.

Several studies point to the need for better regulations for TWA workers with regard to work environment factors and social benefits. In several countries, regulations do not permit employers to dismiss employees during sick leave. For TWA workers these regulations seem to be frequently disregarded [12, 58], which means that TWA workers more often go to work while ill as they otherwise risk unemployment.

Small and medium sized enterprises seem, in most jurisdictions, to be exempted from several legal and policy RTW provisions, and subcontracting may lead to unclear responsibilities for the disabled worker. Many employers in SME’s lack information and knowledge concerning WDP and RTW. An intermediary or network organization providing consulting and support for SME employers, as well as employees, may be one way to improve the conditions for WDP in SME’s. The effectiveness of different forms of governmental financial incentives for SME’s in hiring people with disabilities or ill health need to be evaluated. Future research on WDP interventions need to consider differences between different types of SMEs regarding type of business, effects of social proximity between employer and worker on safety attitudes, and how to develop WDP literacy. Larger studies including a number of SMEs are needed, as many reports are based only on single case studies.

Future research also needs to consider differences between different types of temporary employment contracts as well as duration of temporary employment with regard to work disability prevention and possibilities for return to work after sick leave. In order to improve understanding of how to develop and implement WDP and increase possibilities for RTW, it is important to explore reasons for being employed through a temporary employment contract (is it voluntary, due to ill health and reduced work ability, due to current family or life situation, due to lack of education or language skills?).

The employer perspectives and short- and long-term economic consequences of disability and interventions for WDP and RTW in these special workplaces need to be further elucidated within different social insurance jurisdictions. The limited amount of research on WDP in these settings is, in part, due to difficulties in getting adequate number of respondents for robust quantitative multilevel studies. Quantitative research with subgroup analyses to elucidate conditions for various groups, e.g. core and peripheral workers at a workplace, may be possible. To elucidate the interplay between organizational aspects and workplace conditions, multilevel analyses would be necessary, although this also requires large sample sizes. Qualitative studies do not have these problems. Several of the most applicable cited studies are qualitative, more are needed to better understand which interventions are needed and at which level, i.e. individual level, workplace level, organizational level, or system level. An interesting contribution for future research was recently presented by Amick et al. [59] when proposing a more dynamic life course perspective on work and health. Temporal and contextual factors in labor market experiences are of importance for sensitive periods in individual trajectories. For employees in alternate work arrangements may a life course perspective be particularly relevant.

## Conclusions

While the evolving labor market provides job opportunities for workers who have the right work skills and ability to be flexible, this societal change creates new challenges for RTW, especially for vulnerable groups. Employment and labor laws are designed primarily for the traditional, full-time labor force. They thus create RTW difficulties for special categories of workplaces and workers, leaving gaps in regulations and resources within or outside of the workplace that would support disability. Priorities for future research include:

1. Investigate and compare work disability prevention and return to work aspects of temporary employed, employees in SMEs, teleworkers and lone workers in terms of access to occupational health care and rehabilitation, social context, and influence on work conditions in different jurisdictions.
2. Determine whether, how, and to what extent the approach to RTW for temporary workers according to the Dutch model [18] or the IPS model may be adapted and generalized to other countries and temporary workers.
3. For smaller workplaces, study ways to facilitate communication, establish effective accommodations, and provide ongoing supports when the employer may have little or no experience with work disability. The concept of shared resources among SME’s, and on-call expertise, are worthy of development and formal evaluation.
4. Conduct information on risk factors in these special working populations, and provide evidence of what is effective to prevent work disability and enhance RTW in lone and virtual workers.
5. Research ways to facilitate productive employment of persons with disabilities or those recovering from an illness, as well as the sorts of workplace accommodations that enable workers in alternate work arrangements with disabilities and sickness absence to have a safe and sustained return to work.
